# Multifaceted roles and regulation of nucleotide-binding oligomerization domain containing proteins

**DOI:** 10.3389/fimmu.2023.1242659

**Published:** 2023-10-05

**Authors:** Charneal L. Dixon, Amy Wu, Gregory D. Fairn

**Affiliations:** ^1^ Department of Pathology, Dalhousie University, Halifax, NS, Canada; ^2^ Department of Biochemistry, University of Toronto, Toronto, ON, Canada; ^3^ Department of Biochemistry and Molecular Biology, Dalhousie University, Halifax, NS, Canada

**Keywords:** NOD1, NOD2, Crohn’s, inflammation, peptidoglycan, NF-κB

## Abstract

Nucleotide-binding oligomerization domain-containing proteins, NOD1 and NOD2, are cytosolic receptors that recognize dipeptides and tripeptides derived from the bacterial cell wall component peptidoglycan (PGN). During the past two decades, studies have revealed several roles for NODs beyond detecting PGN fragments, including activation of an innate immune anti-viral response, NOD-mediated autophagy, and ER stress induced inflammation. Recent studies have also clarified the dynamic regulation of NODs at cellular membranes to generate specific and balanced immune responses. This review will describe how NOD1 and NOD2 detect microbes and cellular stress and detail the molecular mechanisms that regulate activation and signaling while highlighting new evidence and the impact on inflammatory disease pathogenesis.

## Introduction

A wide diversity of commensal organisms and opportunistic pathogens constantly interact with the human immune system. The success of the innate immune response to these stimuli relies on the recognition of conserved pathogen-associated molecular patterns (PAMPs) by various pattern recognition receptors (PRRs), and when needed the subsequent initiation of an inflammatory response to promote clearance of infection. Among the PRRs, the nucleotide-binding oligomerization domain (NOD)-like receptors (NLR) provide intracellular surveillance through the detection of cytoplasmic PAMPs and endogenous products of tissue injury termed damage-associated molecular patterns or DAMPs (1, 2). Once activated by their cognate ligand, NLRs mediate host responses via signal transduction mechanisms, including stimulation of nuclear factor kappa light chain enhancer of activated B cells (NF-κB), stress kinases, interferon regulatory factors, inflammatory caspases, and autophagy (1, 3, 4). In this review, we focus on NOD1/NLRC1 and NOD2/NLRC2 – two seminal members of the NLR family – and describe molecular mechanisms that regulate activation and signaling. This includes a discussion of host responses, including ER stress induced inflammatory responses, and the impact of signaling on inflammatory disease pathogenesis.

## The structure of NOD1 and NOD2 receptors

**Figure 1:**
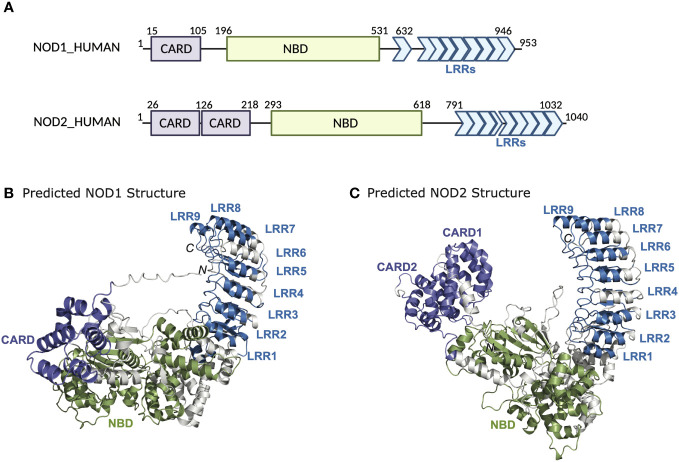
Domain architecture and structure of NOD1 and NOD2. **(A)** Schematic representation of NOD1 and NOD2 highlighting the domain boundaries and amino acid stretches of the CARD, NBD, and LRRs. AlphaFold structures of **(B)** NOD1 and **(C)** NOD2.

**Figure 2:**
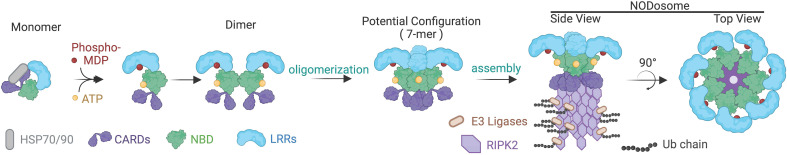
Putative assembly for the NOD2 containing NODosome. Interactions with heat shock proteins, HSP70 and HSP90, stabilize monomeric NOD2. Binding to its ligands ATP via the NBD domain and phosphorylated MDP to the LRR results in conformation changes transitioning from a closed to an open state. Ligand-bound NOD2 is then predicted to assemble into a 7-mer or 8-mer base structure that then serves as a platform for RIPK2 recruitment and poly-ubiquitination, resulting in the functional NODosome. NOD2 is used in this illustration, but it is presumed that a similar activation pathway occurs with NOD1.

NOD1 and NOD2 belong to an evolutionarily conserved family of innate immune receptors characterized by a tripartite domain structure that includes a *C*-terminal domain comprising a variable number of leucine-rich repeats (LRRs), a centrally located nucleotide-binding NACHT domain (NBD), and a variable *N*-terminal effector domain (**Figure 1**). This variable *N*-terminal domain is used to distinguish NLRs into four subfamilies; a) NLRA possesses an acidic transactivation domain, b) NLRB possesses a baculovirus inhibitor of apoptosis repeat (BIR) domain, c) NLRP possesses a pyrin domain, and d) NLRC possesses a caspase activation and recruitment domain (CARD) (5). Members of the NLRC subfamily include NOD1 (*NLRC1, CARD4, CLR7.1*) and NOD2 (*NLRC2, CARD15, CD, BLAU, IBD1, PSORAS1, CLR16.3*). NOD1 and NOD2 have similar domain architecture but differ in the number of CARD domains – NOD1 has one CARD domain, whereas NOD2 has two in tandem. The CARD domain is the region responsible for interactions with the downstream effector kinase receptor-interacting serine-threonine kinase 2 (RIPK2) (6–8). The NBD portion of the proteins mediates self-oligomerization following activation and contains Walker-A and -B box motifs that are important for ATP binding and hydrolysis (9, 10). The binding of ATP to NOD2 enhances both ligand binding and oligomerization, while disruption of the ATP binding domains abrogates muramyl dipeptide (MDP) stimulated signal transduction and NF-κB activation (**Figure 2**) (9, 10). The LRRs constitute the remaining portion of the NODs and are critical for ligand specificity and binding (11).

Available structural evidence suggests that in the absence of ligands, NOD1 and NOD2 exist as monomers in an autoinhibited closed state i.e. ‘folded onto themselves’, producing hairpin-like structures whereby the LRR domain masks the NBD and CARD domains to prevent aberrant signal transduction (12–14). Upon binding of their ligands through the LRRs, it is thought that NOD1 and NOD2 undergo a conformational change that “opens” the protein exposing Walker-A and -B boxes to allow ATP binding, homo-oligomerization and subsequent signaling events (**Figure 2**) (15). However, other structural studies have suggested that the exchange of ADP for ATP, rather than ligand binding, induces structural changes in the NBD domain to shift the state of the protein from inactive to active and *vice versa* (9, 10). This presumes that binding to bacterial ligands is merely a regulatory mechanism to maintain the proteins in an open, active conformation (12). Currently, in the absence of additional structural data to demonstrate how ligand binding could lead to oligomerization, it is impossible to clarify the finer molecular dynamics of NOD1 and NOD2 activation. Furthermore, how membrane association or additional binding partners may influence conformational states and oligomerization are currently unclear.

## Activation of NOD1 and NOD2 by peptidoglycan components

NOD1 is ubiquitously expressed in cells (16), whereas NOD2 has been found primarily in immune (e.g., macrophages, dendritic cells) and epithelial (e.g., Paneth cells, keratinocytes) cells (17, 18). Nevertheless, both proteins play crucial roles in host defense and survival primarily by conferring responsiveness to specific muropeptides present in peptidoglycan (PGN) (19, 20). PGN is a major component of the Gram-positive bacterial cell wall, while in Gram-negative bacteria, PGN comprises only a thin layer in the periplasmic space. Muropeptides are monomeric fragments that can be generated from the degradation of PGN by either host or bacterial enzymes (21). NOD1 activity is primarily triggered by muropeptides containing the minimal core structure γ-D-Glu-*m*-diaminopimelic acid (iE-DAP) (22), that is found in Gram-negative bacteria and a few Gram-positive bacteria, such as *Listeria monocytogenes* and *Bacillus* spp. In contrast, NOD2 detects and directly binds MDP (23), a motif broadly expressed in Gram-positive and Gram-negative bacteria. Although several studies have reported a direct interaction between NOD1 and NOD2 with their respective ligands, it remains to be determined if other cofactors and accessory proteins are involved in ligand binding. In this regard, a recent study has determined that MDP is phosphorylated by a cytosolic host kinase N-acetyl-D-glucosamine kinase (NAGK). Importantly, this study also demonstrates that phospho-MDP is the cellular substrate for NOD2 and NAGK knockout macrophages fail to respond to MDP (24).

**Figure 3:**
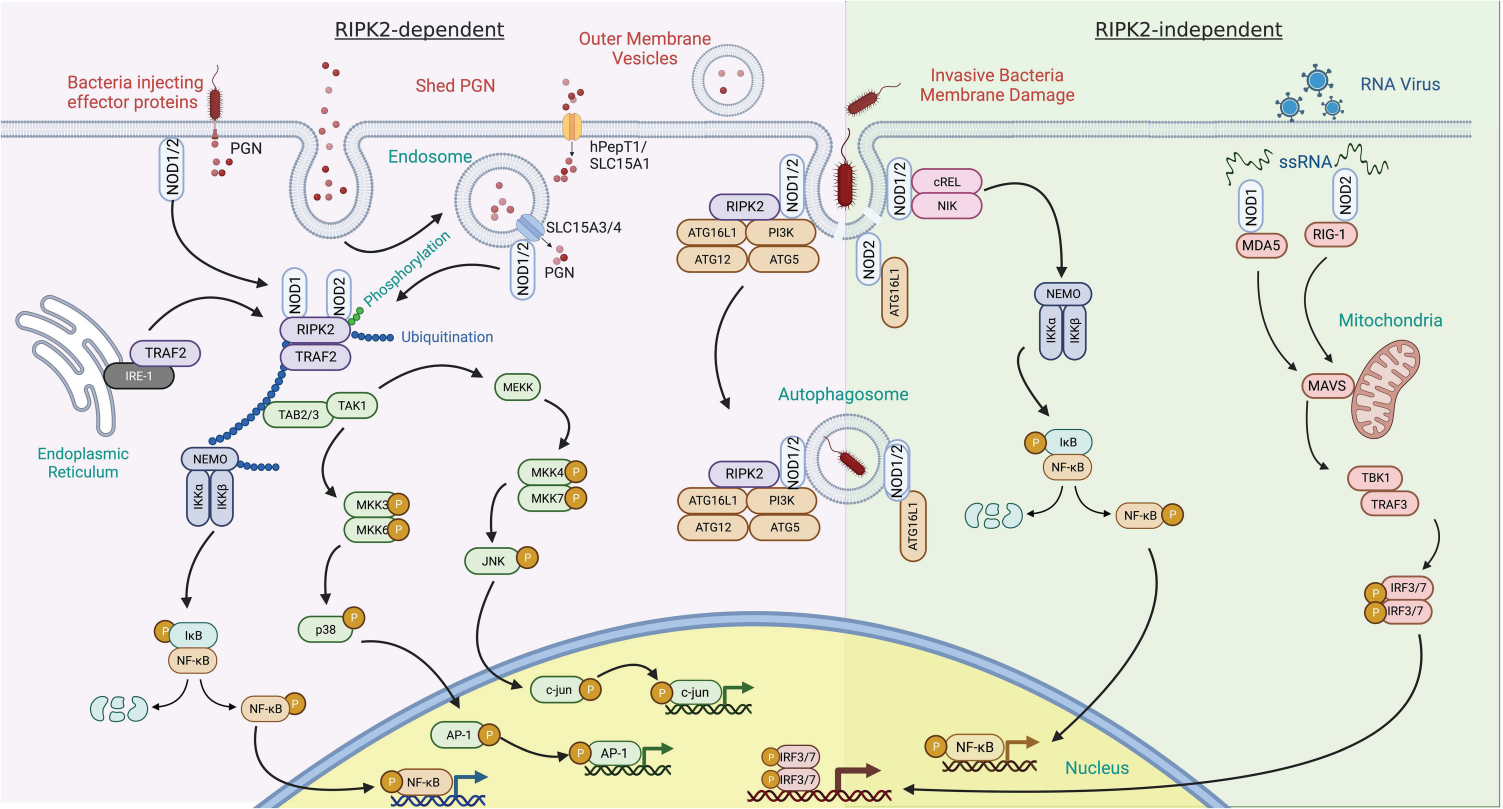
RIPK2 dependent and independent signal transduction in response to pathogen-associated molecular patterns. RIPK2-dependent – activation of NOD1 and NOD2 by peptidoglycan (PGN) components typically leads to recruitment of RIPK2. PGN can enter the cytosol via several mechanisms. This includes co-delivery with injected bacterial effector proteins or the fusion of bacterial outer membrane vesicles with host cells. PGN shed by bacteria can also be delivered to the cytosol by a variety of solute carrier channels (SLC15A family members) residing in the plasma membrane, endosomes, and phagosomes. Binding of RIPK2 to NOD1 and NOD2 results in its phosphorylation and polyubiquitination by TRAF2 and other E3 ligases. This, in turn, can result in the polyubiquitination of NEMO, leading to NF-κB phosphorylation and transit to the nucleus. Tab2/3 and Tak1 recruitment and activation transduces a signal to map kinase family members, further potentiating pro-inflammatory gene induction through c-Jun and AP-1. Invasive bacterial species can induce damage to the bacteria containing vacuole and release PGN. This event has been described as recruiting NOD proteins and the autophagy scaffold ATG16L1. RIPK2-independent – this response is described as occurring in the absence of RIPK2. In addition to PGN, NOD1 and NOD2 bind to ssRNA from RNA viruses and induce upregulation of interferon through MAVS and the transcription factors IRF3 and IRF7.

In contrast with Toll-like receptors (TLRs), which are integral membrane receptors that recognize ligands at the cell surface or in the lumen of endosomes, NOD1 and NOD2 are cytoplasmic microbial sensors that survey for PGN after its transport across the plasma membrane. There are multiple mechanisms by which muropeptides derived from PGN can gain entry to the cytoplasm to activate NOD1 and NOD2, reviewed extensively elsewhere (**Figure 3**) (17, 25). Briefly, PGN or its monomeric muropeptides can be transported across the plasma membrane or limiting membrane of phagosomes/endosomes by peptide transporters SLC15A family members (26, 27). In these situations, bacteria may be internalized into host cells through phagocytosis or bacterial invasion (28, 29). Extracellular PGN shed from bacteria can also be internalized via endocytosis or micropinocytosis (30, 31), or outer membrane vesicles derived from bacteria can serve as a carrier for PGN (32, 33). Alternatively, some instances do not require the transport of muropeptides across host membranes. This includes intracellular bacteria, such as *Listeria monocytogenes*, that gain access to the cytosol and shed muropeptides directly (34), or bacteria that use their secretion systems to breach host membranes and deliver effectors, and PGN, into the cytosol of host cells, as is the case with activation of NOD1 by *Helicobacter pylori* (35). Thus, peptide transporter-dependent and -independent mechanisms contribute to the delivery of muropeptides to the cytosol.

## NOD initiated signal transduction pathways

### RIPK2-dependent signaling – the NODosome

Structural studies have revealed that oligomerization of NOD receptors and subsequent engagement of RIPK2 brings individual RIPK2 molecules into proximity to each other (**Figure 3**) (15). This promotes homotypic interactions of the CARD domain of RIPK2 required for the activation of the canonical NF-κB pathway (15). Specifically, RIPK2 contains both *N-* and *C-*terminal CARD domains that bind with the CARD domains of NOD1 and NOD2 to form a hetero-CARD complex. This promotes RIPK2 polymerization that not only results in a long filamentous assembly that is presumably the core of the NODosome (36), but is also required for Lys63-linked polyubiquitination of RIPK2. Polyubiquitination of RIPK2 can be accomplished by several E3 ubiquitin ligases including TRAF6, XIAP, cIAP1/cIAP2, ITCH, PELLINO3, and LUBAC (37–42). Regardless of the identity of the E3 ligase, the polyubiquitination enables the recruitment of downstream effector proteins (43, 44). Critical mediators downstream of polyubiquitinated RIPK2 include transforming growth factor B-activated kinase 1 (TAK1) and TAK1-binding protein (TAB) to mediate ubiquitination-dependent signaling (7, 45). RIPK2 also interacts with NK-κB essential modulator kinase (NEMO), the regulatory subunit of the IκB kinase (IKK) complex. The simultaneous recruitment of TAK1 and NEMO promotes IKK-mediated phosphorylation of the NF-κB inhibitor IκBα subunit (15), which results in its own polyubiquitination and subsequent proteasomal degradation. This allows the cytoplasmic release and translocation of NF-κB to the nucleus to influence the transcription of proinflammatory cytokines and mediators (45) (**Figure 3**). RIPK2-TAK1 is also an activator of MAPK cascades. Phosphorylation of MKK6 by the TAK1 complex leads to activation of MAPKs, including p38, ERK (extracellular signal-regulated protein kinase), and JNK (c-Jun N-terminal kinase) (46–51). Phosphorylated MAPKs translocate into the nucleus and phosphorylate AP-1 transcription factors that further promote cytokine, chemokines, and antimicrobial peptide expression (52). Thus, the oligomerization of NODs and the subsequent engagement of RIPK2 and other downstream effectors results in key antimicrobial transcriptional responses.

Both NF-κB and MAPK activation has the capacity to induce the transcription of proinflammatory cytokines and mediators, including antimicrobial peptides. However, the exact mediators are context and cell-type dependent. For example, NOD signaling in antigen-presenting cells produces proinflammatory cytokines such as IL-1β, IL-6, TNFα, IL-10, IL-18, chemokines (IL-8), cell adhesion molecules, and nitrite oxide (22, 53–62). Epithelial cells, however, produce pro-inflammatory mediators such as TNF, IL-6, IL-8, macrophage inflammatory protein 2 (MIP2), and antimicrobial peptides (e.g., β-defensin2) (61, 62), in response to NOD activation. Furthermore, in addition to mediating the induction of various proinflammatory genes in innate immune cells, NF-κB regulates the activation, differentiation, and effector function of inflammatory T cells (63, 64) and activation of inflammasomes (65). Thus, context and cell-specific responses can be initiated by NOD1 and NOD2.

The NODosome has been used to describe the core signaling complex of multiple NODs with RIPK2 (28, 66). Thus, the formation of the NODosome is a critical event required for activating downstream signaling networks that include NF-κB, MAPKs, and the interferon regulatory factors for regulating host defense and tissue homeostasis (6–8, 43, 67, 68). The NODosome acts as a scaffold for several effectors at the site of bacterial entry, activating multiple downstream inflammatory signaling pathways to promote the production of cytokines and chemokines that are ultimately important for pathogen clearance (45, 66, 69–71). Other NLR proteins are known to assemble in larger structures including the NLRP3 scaffolded inflammasome and the Apaf-1 mediated apoptosome (8, 15, 72). Indeed, the NACHT-associated NBD domain is part of the larger ATPases associated with diverse cellular activities (AAA-ATPase) superfamily of proteins that typically form hexamers and heptamers (73) (**Figure 2**). As such, the NODosome is generally believed to also function as a hexameric or heptameric protein complex.

### RIPK2-independent signaling and autophagy

Although PGN can potentiate type I interferon (IFN) responses (74) via a RIPK2-dependent pathway (75–77), NOD1 and NOD2 have also been shown to also induce type I IFN signaling in the absence of PGN stimulation and independent RIPK2 (**Figure 3**) (78, 79). NOD2 responds *in vitro* to viral ssRNA and *in vivo* to viruses that express ssRNA during viral infection (70). This response involves binding of ssRNA to NOD2, followed by translocation of NOD2 to the mitochondria, which fosters interaction with the adaptor protein MAVS (mitochondrial anti-viral signaling) via the CARD and NBD domains of NOD2. This interaction leads to interferon regulatory factor-3 (IRF-3) activation in a TRAF-dependent manner and induction of IFNβ production (70, 80). Although NOD1 was not as responsive to ssRNA as NOD2, it can also induce IFN signaling via MAVS in epithelial cells (81, 82).

NODs have also been described to activate autophagic responses, but whether this absolutely requires RIPK2 is unclear. Both NOD1 and NOD2 can detect intracellular bacteria and activate host responses through an alternate pathway involving the cell’s autophagic machinery. This induction of autophagy depends on the interaction of NODs with the adaptor protein ATG16L1 to promote the lysosomal degradation of the invading microbe. However, NF-κB activation is not required. Studies by Travassos et al. (83), and Cooney et al. (84) demonstrated the NOD-mediated induction of autophagy, albeit by contrasting RIPK2-independent and dependent pathways, respectively. Travassos et al. provide evidence that NOD1 and NOD2 interact with ATG16L1 at the plasma membrane in RIPK2-deficient cells (83). On the contrary, Cooney et al. report that a NOD2-specific autophagic response to PGN requires intact RIPK2 function (84), and subsequent studies have also reported the importance of RIPK2 for NOD-dependent autophagy induction (85–87). Yet, the presence of ATG16L1 has been shown to interfere with the polyubiquitination of RIPK2 and thus limit NOD1- and NOD2-induced NF-κB dependent cytokine expression (88, 89). Collectively, these studies would suggest that NODs can engage ATG16L1 independently of RIPK2 but that, under some circumstances, downstream signaling is still initiated. Given that ATG16L1 can function in both canonical autophagy as well as non-canonical LC3-associated phagocytosis, perhaps some of the discrepancies are that the NOD−ATG16L1 interaction can initiate more than one type of response depending on the context and cell type.

## NODs and an ER stress-induced inflammatory response

The endoplasmic reticulum (ER) contributes to protein homeostasis by regulating protein folding, processing, and transport. The aberrant accumulation of proteins overwhelms the protein folding capacity of the ER, resulting in ER stress (90, 91). The cell responds to ER stress by initiating unfolded protein response (UPR), which is aimed at restoring ER proteostasis by increasing the ability of ER to fold proteins properly, regulating protein translation, and, if all else fails, inducing cell death (92–94). The UPR also contributes to inflammation associated with diseases such as Crohn’s disease (95, 96). Specifically, ER stress induced UPR signaling is coupled with the activation of pro-inflammatory pathways, mediated by NF-κB (96). Upon activation of UPR, the ER stress sensor inositol-requiring enzyme 1α (IRE1α) (97) is phosphorylated, resulting in the recruitment of the E3 ligase TRAF2 and the kinase ASK1 to the ER membrane. The IRE1α/TRAF2/ASK1 complex activates the inhibitor of nuclear factor kappa-B kinase (IKK), which phosphorylates IκB leading to its degradation and release of the p65 subunit (NF-kB/RelA), allowing it to translocate into the nucleus and stimulate transcription of pro-inflammatory genes (98). Thus, the potential for crosstalk or synergy between UPR and NOD signaling is apparent.

**Figure 4:**
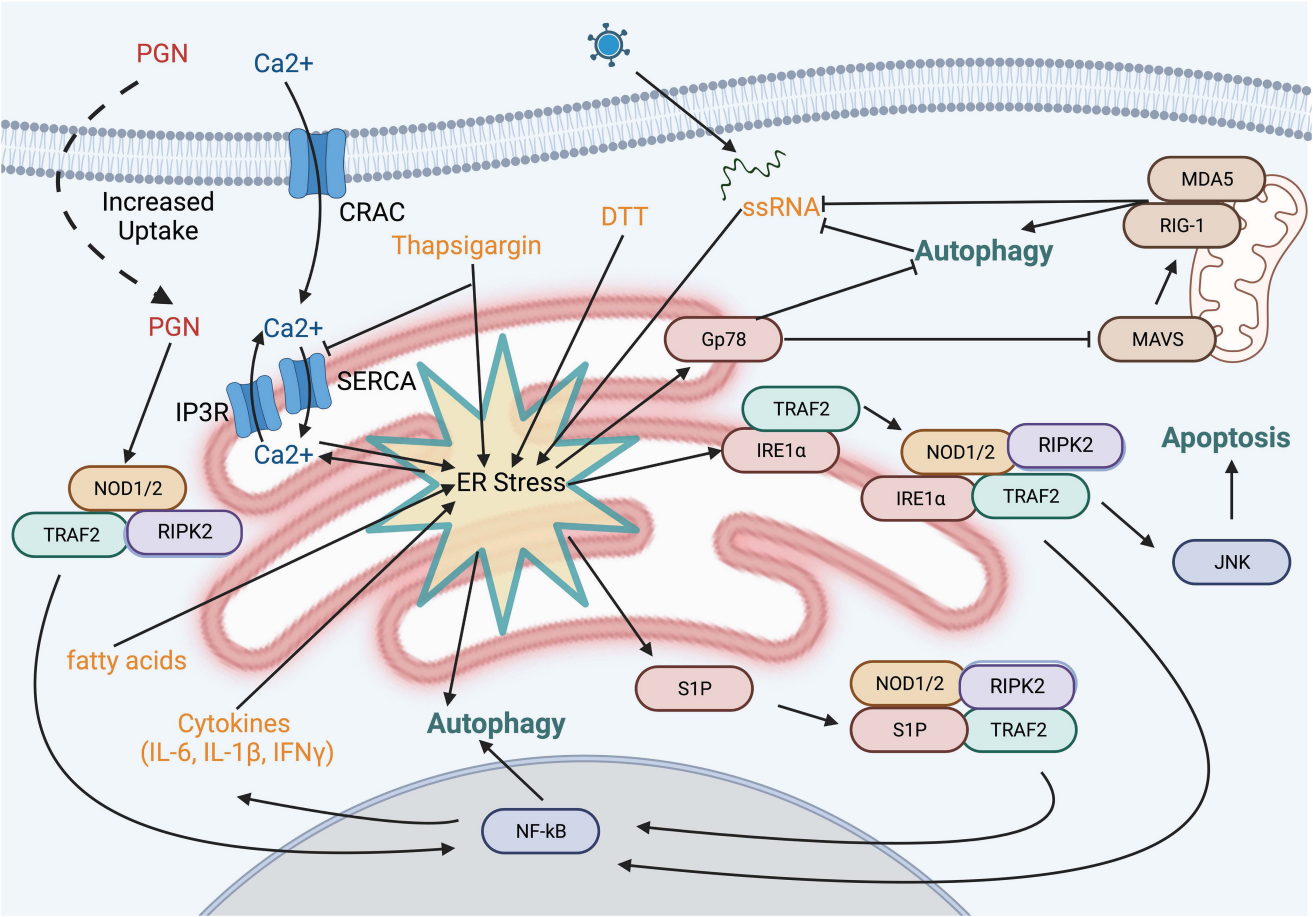
ER stress pathways and NOD activation. Several small molecules and bacterial proteins are known to induce ER stress and activate NOD proteins. Depletion of the ER calcium stores using thapsiagargin, fatty acids, and dithiothreitol is known to activate NOD1/2. However, this may be explained, at least in part, by the sustained increase in cytosolic calcium resulting in enhanced uptake of PGN from the extracellular fluid. Sphingosine 1-phosphate a molecule produced in response to numerous cell stresses, binds to NOD1 and NOD2 leading to upregulation of IL-6 and IL-8.

Beyond the potential convergence of signals, NOD1 and NOD2 have also been implicated in ER stress-induced UPR through IRE1α in a TRAF2- and RIPK2-dependent manner (**Figure 4**) (99). Several chemical inducers of UPR, including dithiothreitol and thapsigargin, induce IL-6 production in a NOD-RIPK2-dependent manner. Additionally, the *Brucella abortus* effector protein VceC induces UPR due to its ability to bind and sequester the ER chaperone BiP (100). Infecting cells with wild-type *B. abortus* resulted in NODs and RIPK2-dependent NF-kB activation, however these proinflammatory responses were notably reduced with the VceC-deficient mutant. Importantly, treating mice and cells with the chemical chaperone t-UDCA was able to reduce ER stress and the NOD-RIPK2 activation of the NF-kB pathway (99, 101). Mechanistically, how NODs sense ER stress in these studies is unclear and has its limitations. Thapsigargin, a molecule that reduces ER luminal Ca^2+^ leading to sustained cytosolic Ca^2+^, resulted in cytokine production in epithelial cells that was only partly dependent on NOD1 and NOD2. However, other ER stress inducers that are not associated with changes in cytosolic Ca^2+^ levels, such as tunicamycin or the bacterial cytotoxin subtilase, that also targets BiP, induced a pro-inflammatory response regardless of NOD1 and NOD2 (102). The authors suggest that rather than UPR directly activating NODs, endocytosis of trace amounts of PGN present in serum stimulates NODs and that this process is potentiated by high cytosolic Ca^2+^ and calcium release-activated channels (102). Indeed, this is a possible confounder in many of the NOD studies as, while vendors routinely report endotoxin levels, they typically do not report levels of PGN or other PAMPs. Still, are either of these mechanisms probable? Could sustained cytosolic Ca^2+,^increase uptake of PGN, and prolong IRE1α activation and TRAF2-mediated polyubiquitination of RIPK2 result in enhanced NF-κB activation? Or could there be yet another signal? In this regard, a general marker and messenger of cell stress, sphingosine-1-phosphate (S1P), was recently shown to bind to the NBD region of both NOD1 and NOD2, triggering downstream NF-κB activation and the induction of pro-inflammatory cytokines (103). Numerous cellular stresses impact a variety of cytoskeletal elements and organelles, resulting in this S1P-mediated response, including the ER stresses tunicamycin and thapsigargin. Whether this response also occurs with DTT and the bacterial effectors VceC and subtilase remains to be examined.

## Post-translational modifications of NOD1 and NOD2

### S-acylation and NOD-mediated immune signaling

**Figure 5:**
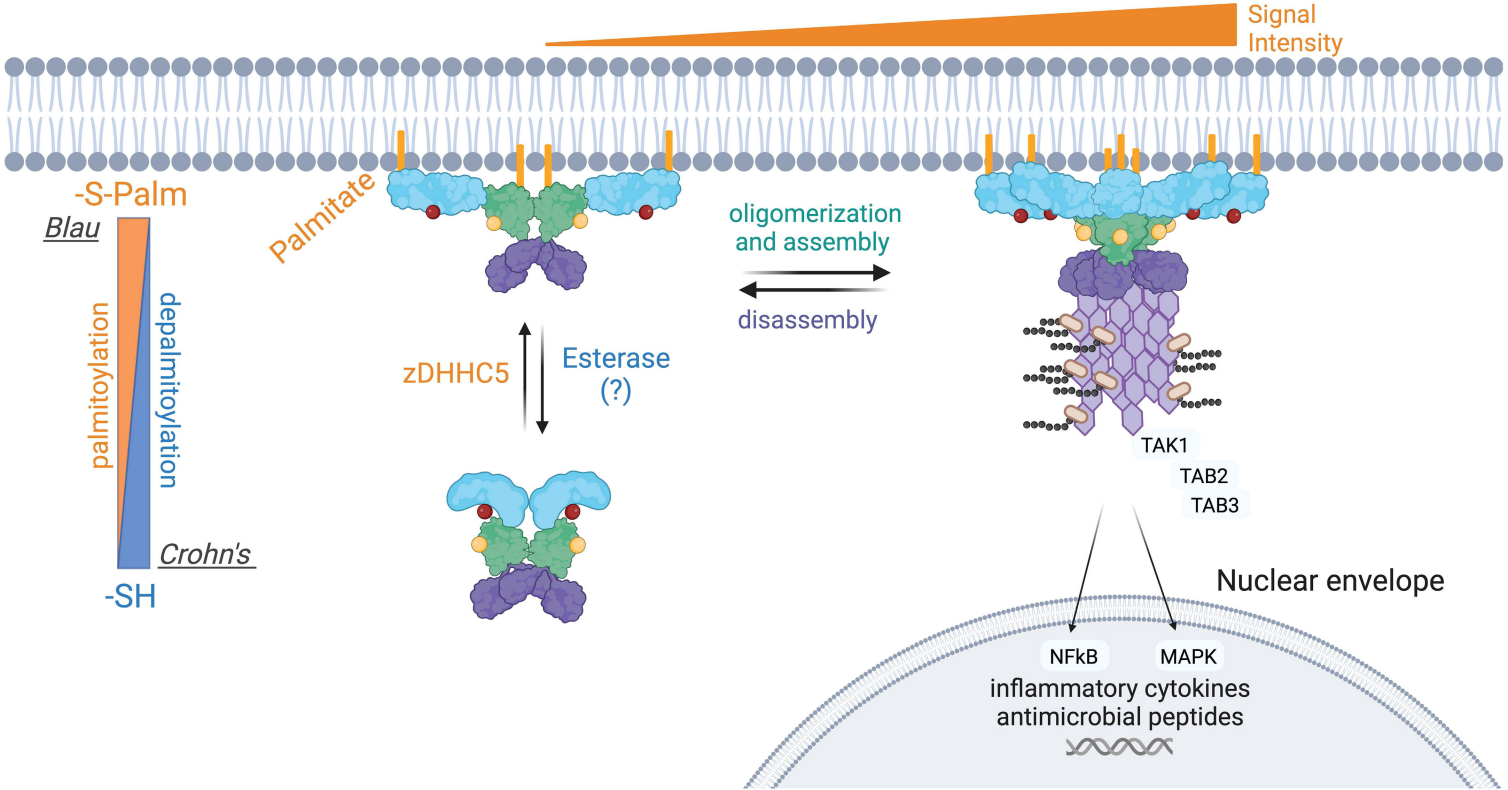
Aberrant S-palmitoylation of NOD2 in Crohn’s disease and Blau Syndrome. Loss-of-function mutations in NOD2 predispose individuals to developing Crohn’s disease, whereas gain-of-function mutations result in Blau syndrome and early-onset sarcoidosis. NOD2 loss-of-function mutations manifests in a variety of ways, including an inability to bind RIPK2 or PGN. NOD2 also contains two essential S-palmitoylation sites for its functionality, and several of the Crohn’s associated NOD2 mutant proteins are hypo-palmitoylated. Conversely, one extensively studied Blau syndrome mutation, NOD2^C495Y^ displays enhanced levels of S-palmitoylation. S-palmitoylation of NOD2, and NOD1, is catalyzed by the protein palmitoyltransferase zDHHC5. S-palmitoylation is a reversible post-translational modification, however the acyl thioesterase(s) that mediate fatty acid removal from NODs have not been identified. The current evidence suggests that both hypo- and hyper-palmitoylation of NOD2 results in aberrant signal transduction.

NOD1 and NOD2 are mainly soluble in the cytosol, yet a small fraction of both is associated with the plasma membrane in unstimulated cells. Upon intracellular infection, both microbial sensors are rapidly redistributed to bacteria-containing phagosomes and endosomal compartments to transduce signals in response to PGN (26, 28, 29, 32). Both proteins lack a transmembrane region and traditional lipid-binding motifs. Instead, the post-translational addition of fatty acids, specifically *S*-acylation, is required for membrane targeting and subsequent signaling in response to PGN detection (104, 105) (**Figure 5**). *S*-acylation of NOD1 was mapped to three cysteine residues Cys558, 567, and 952, whereas NOD2 is modified at Cys395 and 1033 (**Figure 5**). The addition of palmitate or potentially other fatty acids, mediated by the zinc finger DHHC-type palmitoyl transferase 5 (zDHHC5) enzyme, results in NOD proteins with an increased affinity for the plasma membrane. To date, the specific identity of the fatty acid attached to NODs has not been identified but a recent study demonstrated that zDHHC5 has a strong preference of palmitate over other fatty acids tested (106) Cys to Ser mutants of NODs lacking acylation are completely soluble and are unable to induce NF-κB signaling in response to PGN (105, 106). Following the internalization of bacteria, both zDHHC5 and the NODs are recruited to the bacteria-containing phagosomes, a feature absent in the zDHHC5-deficient macrophage (104). Furthermore, the presence of zDHHC5 and SLC15 channels on nascent phagosomes may help NODs locally detect PGN and induce signals.

Still, a complete picture of how NODs are recruited to intracellular membranes remains to be fully elucidated. It was also determined that many human NOD2 mutations associated with Crohn’s disease are inefficiently acylated, explaining their loss-of-function phenotype. Conversely, the C495Y mutant associated with Blau Syndrome displays hyper-acylation in the absence of peptidoglycan while stimulating inflammatory signaling. Restoring the acylation levels of the NOD2 C495Y to baseline reduced the pro-inflammatory signaling in cells. Thus, manipulating the levels of NOD2 acylation could be beneficial for treating Blau syndrome.

### NOD proteostasis – further roles of ubiquitination and chaperones

In addition to *S*-acylation, NOD-mediated signaling is modulated by post-translational ubiquitination. Downregulation of NOD2-induced NF-κB signaling is accomplished through the recruitment of multiple ubiquitin ligases and ubiquitin-editing enzymes. NOD2, but not NOD1, is polyubiquitinated at steady state (107). The E3 ubiquitin ligase TRIM27 binds to NOD2 via the NBD domain and induces K48-linked polyubiquitination, which targets NOD2 for degradation by the 26S proteasome (107), while TRIM22, another E3 ubiquitin ligase, mediates the K63-linked polyubiquitination of NOD2 that is required for the activation of NOD2 signaling, but not its degradation (108). SQSTM1/p62 is an autophagic cargo receptor that recognizes ubiquitinated cargoes, including organelles and protein aggregates, including NOD2 (109). NOD2 *S*-acylation by zDHHC5 attenuates the interaction between NOD2 and cargo receptor SQSTM1/p62. This autophagic degradation of NOD2 is crucial for avoiding an excessive NOD2-mediated response. Ultimately, SQSTM1/p62 provides a negative feedback mechanism for NOD2 signaling and ensures appropriate response during host defense.

The assembly and stability of NOD1/2-RIPK2 complexes require the heme-regulated inhibitor (HIR), an eIF2a kinase, and the associated heat shock protein, HSPB8A to promote the solubility of NOD1 oligomers (110). HSPs are also involved in NODosome assembly (111), and are required for both NOD1- and NOD2-mediated NF-κB activation (112, 113). Genetic silencing and small molecule inhibitor studies demonstrated that HSP90 is important for the stability of NOD1 and NOD2 in MCF-7 cells (114). Furthermore, overexpression of HSP70 increases NF-κB activity following NOD2 ligand-mediated stimulation, whereas HSP70 knockdown reduced NF-κB signaling (115). Analysis of NOD2 protein levels demonstrated that HSP70 acts to stabilize the NOD2 protein, as low HSP70 levels reduced the half-life of NOD2. The association between HSP90 and NOD2 was reproduced by Lee et al. (116), who confirmed its importance for NOD2 stability. HSP90 was suggested to act as part of a negative feedback loop, wherein activation of NOD2 causes its dissociation from HSP90, leading to recognition by SOCS3 and a yet-to-be-identified E3 ligase ultimately leading to proteasome-dependent degradation (116).

Several of the described protein interactions and ubiquitination act as negative feedback mechanisms to fine-tune or attenuate inflammatory signaling. The E3 ligase TRAF4 binds directly to NOD2 to inhibit the activation of NF-κB, creating a negative feedback loop (117, 118). Erbin, an LRR domain-containing protein that is localized to the plasma membrane, binds to NOD2 through an interaction involving the Erbin LRR and the NOD2 CARD domains (119). Downregulation of Erbin enhances MDP-induced NOD2-dependent NF-κB activation, indicating that Erbin is also involved in the negative regulation of NOD2.

### Roles of O-GlcNAcylation in protein stability

*O*-GlcNAcylation of NOD2 is a non-canonical glycosylation that involves the attachment of *O*-*N*-acetylglucosamine (*O*-GlcNAc) moieties to Ser and Thr residues. This dynamic modification is mediated by two enzymes: *O*-GlcNAc transferase (OGT) catalyzes the attachment of *O*-GlcNAc to NOD2, while *O*-GlcNAcase (OGA) hydrolyzes *O*-GlcNAc and returns NOD2 to its unmodified state. Notably, when treated with the OGA inhibitor Thiamet G, NOD2’s *O*-GlcNAcylation levels increased. This elevation had a dual effect: it bolstered the stability of NOD2 and amplified NOD2-mediated NF-κB signaling in response to MDP stimulation (120). Interestingly, Crohn’s disease-associated mutant forms of NOD2, namely R702W and 3020insC, exhibit diminished protein stability. Intriguingly, *O*-GlcNAcylation has been shown to counteract this instability and promote downstream NF-κB activity when subjected to TNF-α and MDP stimulation (120). Like NOD2, *O*-GlcNAcylation also stabilizes NOD1 and enhances its signal transduction capability (121). While *O*-GlcNAcylation has been validated as a mechanism that augments NOD1/2 signaling by fortifying protein stability, the specific *O*-GlcNAcylation sites remain unidentified.

### Small G-proteins activation, actin dynamics and NOD recruitment

Several small Rho family G-proteins Rac1, Ccd42, or RhoA lead to the assembly of the NODosome at the plasma membrane (122). Furthermore, several secreted bacterial effector proteins result in prolonged activation of small Rho GTPases and NOD signaling pathways (112, 119, 123–125). Mechanistically, the Rho family GTPase could facilitate the activation of NODs in two non-exclusive ways. First, the Rho family member could recruit more NOD1/2 to the plasma membrane or endosomes through direct protein interactions. Second, stimulation of Rho family G-proteins can lead to cytoskeletal rearrangement and the subsequent internalization of pathogens or PAMPs into the host cell (126–128). The relative importance of these two possibilities is unclear, yet the activation of the G-proteins in these circumstances leads to proinflammatory responses (129). The membrane localization of small Rho GTPases is essential for their ability to activate the NODosome because a constitutively active form of Rac1 lacking its prenyl-group is unable to activate NOD1 (112). Altogether, this indicates that the association of NODs with Rho GTPases is a possible contributor of NOD recruitment to the plasma membrane. Whether this requires the *S*-acylation of NOD1 and NOD2 or whether the hyperactivation of small G-proteins can bypass this requirement has yet to be determined.

NOD1 is recruited to membrane ruffles by a complex consisting of Rac1, CDC42, and Hsp90 (112, 130). Rac1 and NOD2 co-localize at membrane ruffles, while Rac2 and NOD2 colocalize with RIPK2 at ruffles; however, if Rac1 is knocked down, NOD2 membrane localization is abrogated (112, 131–133). Like NODs, the small Rho GTPase Rac2 colocalizes with RIPK2 at membrane ruffles (131, 134). These interactions may reinforce the idea that the actin cytoskeleton recruits NODs as Rac1 modulates actin in cell movement and membrane protrusions (135). NOD2 also colocalizes with GEF-H1, a GEF for RhoA (136). The finding that activation of Rac1/2, Cdc42, and RhoA trigger NOD signaling and NODosome assembly is significant as small Rho GTPases are well-known targets of bacterial virulence factors (137).

Type III secretion systems (T3SSs) of enteric pathogens inject effectors activate small Rho GTPases. Some examples include *Salmonella Typhimurium* inositol phosphatase/phosphotransferase SopB, which activates Cdc42 (138–140), *Escherichia coli* ESpM2 which activates RhoA (141) and Cytotoxic Necrotizing Factor 1 which activates Rac1 (142–144). Furthermore, *Campylobacter jejuni* secretes Campylobacter invasion antigens that ultimately translocated into the cytosol of host cells (145), contributing to the activation of Rac1 (146). The *S. Typhimurium* T3SS effector SipA has actually been shown to activate NOD-dependent NF-κB signaling (133). In a NOD1- and RhoA-dependent manner, *Shigella flexneri* T3SS effectors OspB and IpgB2 induce membrane ruffling resulting in the recruitment of GEF-H1 and downstream NF-κB signaling (147, 148). More specifically, it has been demonstrated that SopE introduces membrane ruffles and recruits the NOD1 into a multiprotein complex containing SopE, Rac1, Cdc42, and Hsp90 at the host cell membrane (112, 130).

## Adjuvant activity of NOD signaling and links to adaptive immunity

Impairment of NOD1 and NOD2 activity in some infections abrogated innate and adaptive immune responses (149, 150). Innate sensing of PGNs by NOD1 primes antigen-specific T cell immunity and the resultant antibody response (151). NOD ligands may also enhance B and T cell activation after the engagement of their respective receptors (152–154). MDP-stimulated NOD2 can drive the production of B lymphocyte chemoattractant in an NF-κB inducing kinase-dependent manner (155). In human tonsillar B cells, iE-DAP or MDP was insufficient to trigger B cell activation or proliferation, but the combination of these ligands and IgM or IgD stimulation resulted in enhanced cellular proliferation and induction of cell surface markers, as well as prolonged survival (152). Peripheral B cells were activated by NOD1 and NOD2 ligands, while tonsillar B cells responded solely to NOD1. CD3+ human tonsillar T cells stimulated with either NOD1 or NOD2 ligands alone or after TCR activation with anti-CD3/CD28 failed to induce cellular proliferation or T-cell cytokine production. However, anti-CD3 stimulation followed by NOD1 ligand did enhance IFNγ production in CD3+ T cells (154).

Both NOD1 and NOD2 promote thymic stromal lymphopoietin protein production and upregulate OX40 ligand surface expression, both promoting Th2 immunity (156, 157). Mice immunized with the NOD1 agonist FK156 and ovalbumin (OVA) demonstrated an enhanced Th2 polarized antigen-specific response (151). Immunization of mice with MDP and OVA results in a Th2 polarized antigen-specific T and B cell response that was NOD2-dependent (156, 158). The NOD2-induced antigen-specific immune response consists of a Th2-type polarization profile that is characterized by IL-4 and IL-5 and IgG1 Ab responses (158).

NOD signaling is also critical for cross-priming in dendritic cells (DCs), as exposure of NOD ligands to mice increased cross-presentation by enhancing antigen presentation and costimulatory molecule expression in DCs (159). Additionally, NOD2 was shown to trigger CD4- DC maturation, and CD4+ DCs showed 10-fold higher NOD2 mRNA than CD4- CDs (160). Additionally, mice deficient in NOD1 (151) or NOD2 (149) exhibited reduced IFNγ levels and decreased antibody production. NOD1^-/-^ mice had reduced amounts of CD4+ and CD8+ cells (151). Similarly, NOD2 ^-/-^ mice showed a lack of CD4+ cells and impaired production of IL2 (149). Furthermore, RIPK2 modulates the activation of CD11c^int^CD11b^+^ DCs in the spleen upon NOD activation (161). adjuvants and their effects on DC maturation markers, yet it is important to acknowledge the extensive body of research on the synergistic interactions between NOD and TLR signaling pathways (162). One compelling example can be seen in the cooperation between NOD2 and TLR2 agonists in inducing CD80, CD83, CD86, and major histocompatibility complex class II (MHC II) molecules in human monocyte-derived DCs (163). However, due to the extensive nature of NOD/TLR interactions and their implications in immunology, a comprehensive discussion of these interactions is beyond the scope of this review.

Moreover, NOD1 and NOD2 stimulation induces an increased expression of MHC II proteins on the surface of APCs (83, 87, 164). NOD2-induced autophagy in DCs is required for MHC-II antigen presentation and antigen-specific CD4+ T cell responses (84). This requires the fusion of autophagosomes with multivesicular MHC class II-loading compartments in APCs (165).

One of the more well-known ways in which NODs contribute to adaptive immunity is the adjuvant activity of MDP and its derivatives. Specifically, muropeptides express strong synergy with other ligands, eliciting a stronger immune response together. NOD2 enhances the production of IgG1-type antibodies to T-cell-dependent antigens (166), which means MDP can be used as an adjuvant to increase antibody production, boosting the potency of therapeutic molecules (167). In this way, muropeptides drive the expression of surface markers necessary for cell adhesion and antigen presentation, increasing phagocytic and anti-pathogenic activity and amplifying cytokine production (168–170). Adjuvant MDP synergizes with IL2, IL4, and LPS (170, 171). Priming mice with MDP increased resistance to microbial infection and enhanced cytokine release (172, 173). Muropeptides increase IFNγ, stimulating the differentiation and proliferation of lymphocytes (170, 171, 174). In fact, pre-stimulation of NOD2 with bacterial ligands improved the ability of dendritic cells (DCs) to prime virus-specific CD8+ T cells in the context of influenza A virus infection (80). Muropeptides and MDP derivatives are also studied to boost immune responses for clinical purposes.

## NODs in inflammatory disease states

NOD1 and NOD2 have been implicated in both acute and chronic inflammatory diseases (2, 19, 20), most recent of which includes Whipple disease (175) and SARS-CoV-2 (176). Mutations and single nucleotide polymorphisms of NOD2 create a genetic predisposition for autoimmune (Crohn’s disease (177, 178)) and autoinflammatory diseases (Blau syndrome (179), Yao syndrome (180), early-onset sarcoidosis (181), and atopic disorders (182)). Genetic variations in NLRC1 (NOD1), though not serving as direct genetic markers of disease, may be more closely associated with an elevated susceptibility to ulcerative colitis, arthritis, asthma, and Behçet’s syndrome (183). Polymorphisms in *NLRC2* (NOD2) are the strongest known genetic risk factors in the development of Crohn’s disease (178). Three common NOD2 variants (R702W, G908R, and 3020insC) are linked to the development of Crohn’s disease (177, 184).

However, the mechanisms by which NOD2 variants contribute to disease pathogenesis remain incompletely understood but involves both genetic and environmental components. NOD2 is highly expressed in Paneth cells and responsible for the expression and secretion of anti-microbial peptides such as β-defensin 2 (185). It is believed that a reduction of antimicrobial peptides causes dysbiosis, which leads to the recruitment of pro-inflammatory immune cells and a shift towards more pathogenic bacteria. To better understand the disease, several *NOD2* loss-of-function mouse lines have been generated to elucidate its role in Crohn’s disease and Blau Syndrome (186–188). *NOD2*-deficient mice display increased susceptibility to bacterial infection while spontaneous intestinal inflammation is consistently absent in *NOD2* ^-/-^ mice or knock-in mice (166, 188, 189). The challenges associated with developing a robust animal model have hindered some of the progress in this field. Instead, there has been a move towards using more patient-derived samples and 3D cell culture models to complement *in vitro* and 2D culture models.

NOD2 mutations associated with Crohn’s disease tend to be confined to the LRR domain and have been shown to abrogate MDP detection and activation of NF-κB in transient transfection experiments (190). Additionally, monocytes from Crohn’s disease patients with the 3020insC frameshift mutation display defects in the secretion of TNFα, IL-6, IL-8, and IL-10 (191, 192). Together these studies suggest that Crohn’s disease-linked mutations result in a loss-of-function phenotype. However, Crohn’s disease has also been associated with the presence of activated NF-κB and inflammatory NF-κB target gene products in epithelial cells and lamina propria macrophages (193, 194), resulting in a protracted controversy as to whether Crohn’s disease-linked mutations in NOD2 diminish or enhance its activity in the context of the disease.

Blau syndrome is associated with apparent NOD2 gain-of-function mutations rather than a loss-of-function typically observed for Crohn’s disease. In the context of Blau syndrome, NOD2 mutations are primarily confined to the NBD region, resulting in an overactivation of the NOD2 phenotype (179, 195, 196). These mutations have been shown to increase NF-κB activity independent of MDP (197). However, complementary mutations in NOD1 do not reflect the same phenotypic expression, suggesting that activation and regulation of NOD1 and NOD2 proceed by distinct mechanisms (196). While most NOD2 mutations associated with Blau syndrome development are thought to be gain-of-function, there are also reports pointing to NOD2 loss-of-function in Blau syndrome development. Notably, in animal model experiments, mice carrying the Blau syndrome R314Q mutation do not develop the disease but instead, show decreased MAPK and NF-κB activation and reduced levels of circulating inflammatory cytokines despite MDP stimulation (187).

Yao syndrome, formerly broadly referenced as NOD2-associated autoinflammatory disease, is a genetically complex multifactorial disease characterized by periodic fever, dermatitis and inflammatory arthritis and gastrointestinal symptoms without inflammatory bowel disease (198). Recently, specific NOD2 variants (IVS8 + 158 and R702W), have been linked to Yao syndrome (70, 180, 199). Other rarer NOD2 variants have also been identified at a low frequency in the disease (200) and several of these NOD2 variants are common to CD and have been demonstrated to impair NOD2 function *in vitro* (184, 200, 201). Autoinflammatory diseases like Yao disease may remain silent until an exogenous trigger activates the pathway (199, 202, 203), and are often polygenetic and may require several mutations to act in concert. While NOD2 variants are a characteristic genotypic feature of Yao disease, it remains unclear how NOD2 dysfunction influences inflammation or disease progression.

Circulating monocytes from patients with type 2 diabetes have increased expression of both NOD1 and NOD2 as well RIPK2 and NF-κB (204). Feeding mice a high-fat diet (HFD) used to mimic a Western diet, results in weight gain and upregulation of both *NOD1* and *NOD2* (205, 206) and mice on an HFD regimen also induces insulin resistance and chronic low-grade inflammation. The *NOD1/2* ^-/-^ double knockout (DKO) mice, when fed an HFD, were protected from many of the detrimental effects, including inflammation, lipid accumulation, and peripheral insulin resistance (207).

Given that NOD1 and NOD2 have different ligands and tissue distribution, subsequent studies interrogated the role of each individually. Like the DKO, the *NOD1* ^-/-^ mice were resistant to the development of HFD-induced metabolic syndrome (208), and this effect was mediated by both hematopoietic and non-hematopoietic cells (209). Further support for the role of NOD1 in response to the HFD diet was obtained by injecting mice with the NOD1 ligand tetra-DAP, which was sufficient to cause whole-body insulin resistance and reduced glucose clearance (208). Importantly, this NOD1 activation and NOD2 activation effects were lost in RIPK2-lacking animals (210).

In contrast, to the role of NOD1 in promoting metabolic syndrome, NOD2 was demonstrated to counteract it. For instance, the *NOD2* ^-/-^ displayed both increased inflammation and impaired insulin signaling in response to HFD (211). Alternatively, both a prophylactic and treatment regimen of MDP in HFD fed mice improved insulin sensitivity and glucose tolerance (212). As mentioned in the previous paragraph, the NOD2 effect required functional RIPK2 and was mediated in part by the transcription factor IRF4 (212) which is known to have anti-inflammatory pathways in the liver and adipose tissues (213). However, like Crohn’s disease, dysbiosis and impaired barrier function in animals without NOD2 allow bacteria to disseminate from the gut to the liver and adipose tissue (211), a feature that has been recapitulated using biopsy samples including liver, various adipose tissue, and plasma in persons with type 2 diabetes (214). Collectively, these results highlight the importance of microbe sensing, barrier function, inflammation, and metabolism. Remarkably, a recent study has also demonstrated that NOD2 activation can support insulin production and signaling in undernourished infant mice, adding yet another layer of complexity to this system (215).

## Concluding remark

In the 20+ years since the discovery of NOD1 and NOD2, various studies have highlighted both their importance and the complexity of these proteins. The inherent complexity of these proteins and their signal transduction pathway is found not only at the protein level but also at the cellular and whole-body levels, including the shaping of the microbiome. Many questions remain unresolved, including the structural regulation and assembly of the NODosome, the relative importance of the numerous protein binding partners and how NODs may contribute to sensing ER stress and the UPR. Furthermore, while much of the work has been studied from the perspective of PGN detection and signaling, the specific roles of NODs in other pathways and potential crosstalk with other inflammatory and anti-viral pathways remains a fruitful area for further study.

## Author contributions

CD and AW contributed equally to the writing of this manuscript and the creation of figures. GF oversaw the project, edited figures, revised the text, obtained funding. All authors contributed to the article and approved the submitted version.
